# Multiple breath washout test data for healthy controls

**DOI:** 10.1016/j.dib.2020.106641

**Published:** 2020-12-10

**Authors:** Lauren M. Zell-Baran, Silpa D. Krefft, Camille M. Moore, Jenna Wolff, Richard Meehan, Cecile S. Rose

**Affiliations:** a National Jewish Health, Division of Environmental and Occupational Health Sciences, 1400 Jackson St., Denver, CO 80206, United States; b Veterans Administration Eastern Colorado Health Care System, Division of Pulmonary and Critical Care Medicine, Aurora, CO, United States; c National Jewish Health, Center for Genes, Environment and Health, Denver, CO, United States; d University of Colorado, School of Medicine, Aurora, CO, United States; e University of Colorado, Department of Biostatistics and Informatics, Aurora, CO, United States

**Keywords:** Multiple breath washout, Lung clearance index, Healthy control, Lung function

## Abstract

This article includes pulmonary function data collected via multiple breath nitrogen washout for 103 healthy U.S. adults recruited at National Jewish Health in Denver, Colorado. Testing was performed by certified technicians and reviewed by expert pulmonologists for quality and consistency. Data were collected from a diverse population that included 52 males and 51 females with an average age of 39 years (range 20–77 years). Participants were of non-Hispanic White (85%), African-American/Black (6%), Hispanic (4%), more than one race (4%) or American Indian/Alaskan Native (1%) race/ethnicity. The majority were never smokers (85%), but 12% were former smokers and 3% were current smokers. Height, weight, and body mass index (BMI) were collected in addition to multiple breath washout (MBW) test parameters such as the lung clearance index (LCI) score.

## Specifications Table

**Table:** 

Subject	Pulmonary and Respiratory Medicine
Specific subject area	Pulmonary physiology measurements using multiple breath nitrogen washout testing
Type of data	Table Figure
How data were acquired	Instrument: Eco Medics AG Exhalyzer D system with adult setup and oxygen tracer gas Software: Spiroware by Eco Medics AG
Data format	Raw
Parameters for data collection	Sex Race Ethnicity Age (years) Height (cm) Weight (kg) Smoking status Smoking pack-years Functional Residual Capacity (FRC) (L) Lung Clearance Index Score (2.5%) Lung Clearance Index Score (5.0%) First Moment of washout curve (M1/M0) Second Moment of washout curve (M2/M0) Scond * Tidal Volume (Scond*VT) Sacin * Tidal Volume (Sacin*VT) Respiratory Quotient (RQ) Tidal volume/FRC (VT/FRC) Mean VT (mL) VdCO2 (mL) Cumulative Expired Volume (CEV) (L)
Description of data collection	Healthy control status was assessed based on self-reported respiratory symptoms and diagnoses. Control subjects were screened via pre-bronchodilator spirometry before undergoing multiple breath washout testing, and excluded if they reported symptoms, lung diagnoses or had abnormal spirometry. Testing was performed during a single study visit by trained technicians who were certified by SickKids Hospital in Toronto, Canada for MBW and by the National Institute for Occupational Safety and Health for spirometry.
Data source location	Institution: National Jewish Health City/Town/Region: Denver, CO Country: United States of America
Data accessibility	With the article (see “Control_MBW_data.xlsx”).
Related research article	Zell-Baran, L., Krefft, S.D., Moore, C.M., Wolff, J., Meehan, R., Rose, C.S. Multiple Breath Washout: A Noninvasive Tool for Identifying Lung Disease in Symptomatic Military Deployers. Respir. Med. In Press. [3]

## Value of the Data

• Published normative data for healthy adults using multiple breath washout is limited and has not included a heterogeneous population.
• Investigators working with adult lung disease patients will benefit from access to this data as a reference for healthy multiple breath washout values.
• Others can build upon this data to create an even more robust set of normative data to be used consistently across study sites.

## 1 Data Description

**Fig. A1:**
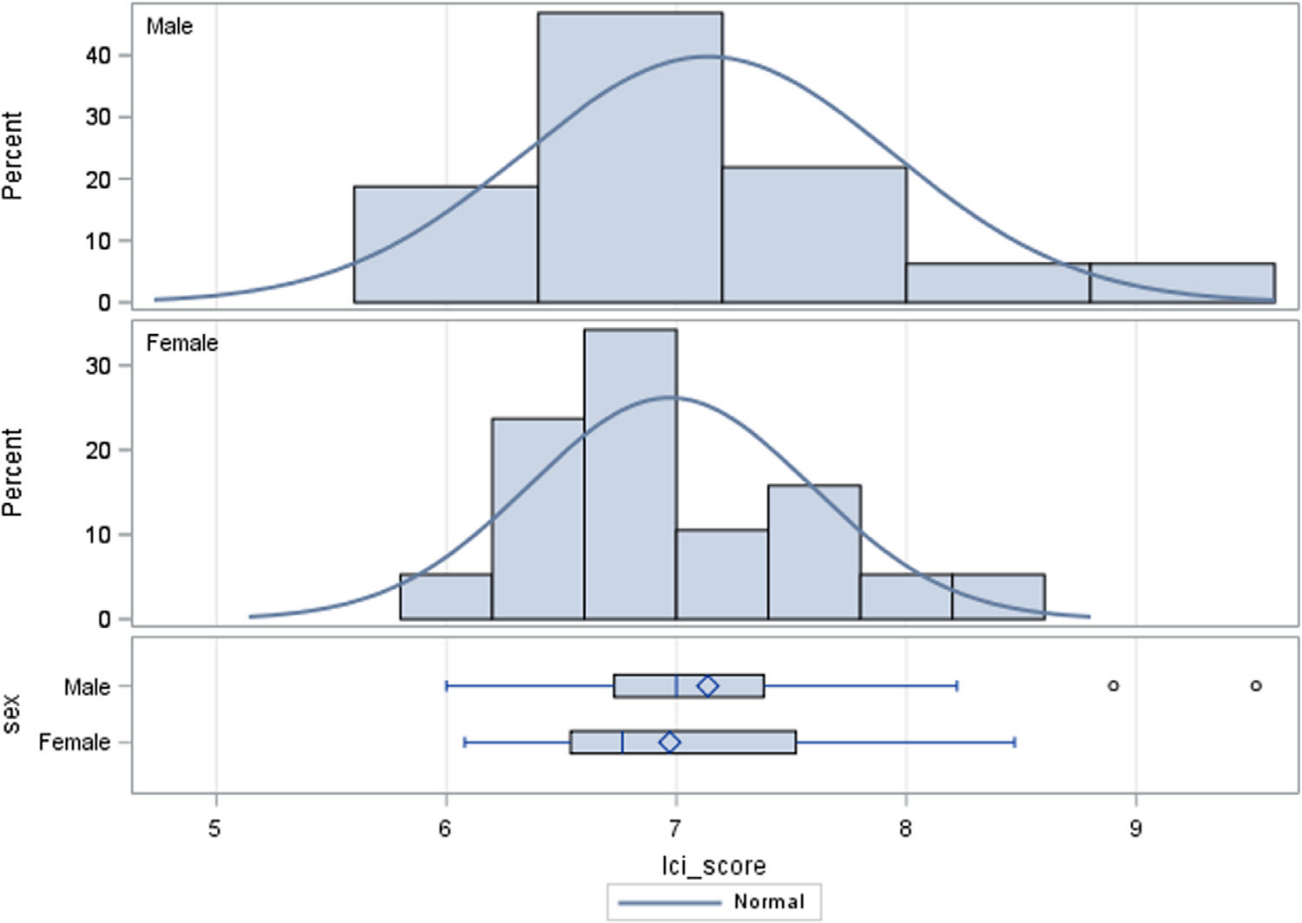
Distribution of lung clearance index scores among never smoking controls with body mass index <30 by sex.

**Fig. A2:**
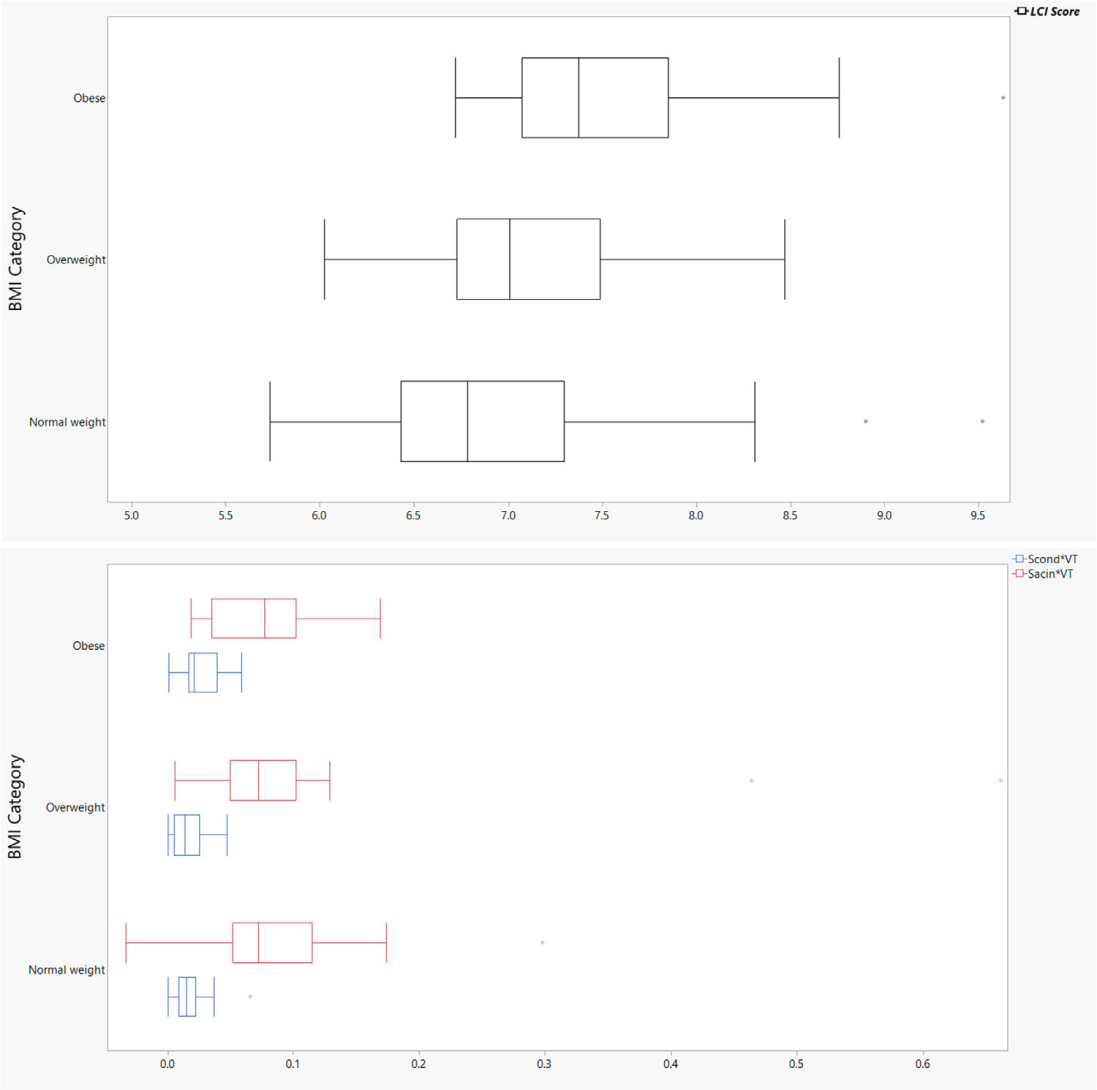
Distribution of multiple breath washout parameters by body mass index category

Histograms of LCI scores among never-smoking controls with BMI values <30 (*n* = 70) are presented in Fig. A1. The upper limit of normal (ULN) was calculated as the mean + 1.96*standard deviation [1]. Among males (*n* = 32), the ULN was 8.71 and among females, the ULN was 8.17. We categorized BMI as normal weight (BMI<25), overweight (BMI25–29.9), or obese (BMI>30) [2] in Fig. A2 to display the relationship between BMI and MBW parameters.

**Raw Data:** Raw multiple breath washout data and relevant demographic characteristics are included on the attached Excel file for download. Data from all trials are not provided, rather results are the average of all acceptable trials. A description of the variables in the raw dataset is presented in Table A1.

**Table A1:** Description of variables in raw dataset.

Variable Name	Description	Units or Levels
sex	Sex	Male, Female
race	Race	African-American/Black, American Indian/Alaska Native, More than once race, White
ethnicity	Ethnicity	Hispanic, Non-Hispanic White, Other
age	Age	years
height_cm	Height	cm
weight_kg	Weight	kg
bmi	Body Mass Index	kg/m2
smoking_status	Smoking status	Never, Former, Current
packyears	Smoking pack-years	pack-years
frc	Functional Residual Capacity (FRC)	L
lci_score	Lung Clearance Index Score (2.5%)	unitless
lci_5_norm	Lung Clearance Index Score (5.0%)	unitless
m1_m0	First Moment of washout curve (M1/M0)	unitless
m2_m0	Second Moment of washout curve (M2/M0)	unitless
scondvt	Scond * Tidal Volume (Scond*VT)	unitless
sacinvt	Sacin * Tidal Volume (Sacin*VT)	unitless
rq	Respiratory Quotient (RQ)	unitless
vt_frc	Tidal volume/FRC (VT/FRC)	unitless
vtmean_ml	Mean VT	mL
vd_co2	VdCO2	mL
cev	Cumulative Expired Volume (CEV)	L

## 2 Experimental Design, Materials and Methods

Participants were recruited at National Jewish Health in Denver, Colorado between March 2015 and March 2020 as part of a larger study aimed at understanding deployment-related lung diseases. Participants were screened for both previously diagnosed chronic respiratory disease and for acute respiratory illness in the four weeks preceding scheduled testing. All testing was completed in a clinical research testing room with medical air and oxygen hook ups. To confirm healthy control status, pre-bronchodilator spirometry was performed 15 min before multiple breath washout testing using American Thoracic Society Guidelines [4,5]. Participants with forced vital capacity percent predicted (FVCPP), forced expiratory volume in one second percent predicted (FEV1PP), or FEV1/FVC ratio below the lower limits of normal (LLN) based on published reference values were excluded from MBW testing. [6] 25 of the subjects were from the pilot study and did not have spirometry, which was added to data collection after the pilot.

MBW testing was performed using the Eco Medics AG Exhalyzer D system and Spiroware software. On each day of testing, environmental settings including temperature and pressure were adjusted, flow and gas channel calibrations were performed, and signal synchronizations were performed. Adult size filters and mouthpieces were used with dead space reducer set 3 (DSR 3). Spirettes and nafion tubes were replaced at recommended intervals.

During testing, subjects were seated upright, wore a nose clip, and were advised to perform relaxed breathing. Subjects began normal breathing on room air to establish tidal volume. The washout phase used 100% oxygen and concluded when subjects’ nitrogen concentrations were below 1/40 or 2.5% of their initial concentration. Results presented are the average of at least two acceptable trials. Trials were deemed unacceptable if 1) the tracer gas did not re-equilibrate between trials, 2) there was clear evidence of a leak, 3) the breathing pattern was erratic, or 4) the trial did not meet end of test criteria described by Jensen et al. [7].

## Ethics Statement

Study participants agreed to complete testing with written informed consent under studies HS-2851 and HS-2985 approved by the National Jewish Health Institutional Review Board.

## CRediT Author Statement

**Lauren Zell-Baran**: Formal Analysis, Investigation, Writing – Original Draft, Visualization. **Silpa Krefft**: Conceptualization, Validation, Writing – Review & Editing. **Camille Moore:** Methodology, Formal Analysis, Writing – Review & Editing. **Jenna Wolff:** Investigation, Project Administration, Writing – Review & Editing. **Richard Meehan:** Conceptualization, Writing – Review & Editing. **Cecile Rose:** Conceptualization, Resources, Writing – Original Draft, Supervision, Funding.

## Declaration of Competing Interest

Cecile Rose receives research grant funding from the U.S. Department of Defense as part of a large multi-site, multi-investigator study on mechanisms of lung epithelial injury. Silpa Krefft is employed by the U.S. Department of Veterans Affairs (DVA) and receives research grant funding from the DVA. Both Drs. Rose and Krefft have participated in medicolegal depositions to provide expert testimony on patients for whom they have rendered medical opinions; however, they have received no personal income or compensation for these medicolegal efforts, all of which have been reimbursed to National Jewish Health.

## Appendix Supplementary Materials

Image, application 1
